# **Evaluation of Probiotic Survivability in Yogurt Exposed To Cold Chain Interruption **

**Published:** 2013

**Authors:** Rohollah Ferdousi, Millad Rouhi, Reza Mohammadi, Amir Mohamad Mortazavian, Kianosh Khosravi-Darani, Aziz Homayouni Rad

**Affiliations:** a Department of Food Technology Research, National Nutrition and Food Technology Research Institute, Shahid Beheshti University of Medical Sciences, Tehran, Iran.; b Department of Food Science and Technology, Faculty of Agriculture, University of Tehran, Karaj, Iran.; c Students Research Committee, Department of Food Science and Technology, National Nutrition and Food Technology Research Institute, Faculty of Nutrition Sciences, Food Science and Technology, Shahid Beheshti University of Medical Sciences, Tehran, Iran.; d Students’ Research Committee, Department of Food Science and Technology, National Nutrition and Food Technology Research Institute, Faculty of Nutrition Sciences, Food Science and Technology, Shahid Beheshti University of Medical Sciences, Tehran, Iran.; e Department of Food Science and Technology, Faculty of Nutrition, Tabriz University of Medical Sciences Tabriz, Iran.

**Keywords:** Bifidobacteria, *Lactobacillus*, Probiotic, Room temperature, Survival, Viability

## Abstract

In this research, the survival of probiotic microorganisms in yogurts stored at room temperature (cold chain interruption conditions) was studied. Milk inoculated with yogurt bacteria (mixed culture of *Streptococcus thermophilus *and *Lactobacillus delbrueckii *ssp. *bulgaricus*) and a single probiotic culture (*L. acidophilus *LA-5 or *Bifidobacterium lactis *Bb- 12 or *L. rhamnosus *HN001 or *L. paracasei *Lpc-37) were incubated till pH of 4.5 was reached. Probiotic yogurts were stored at two different temperatures including cold (control) and room temperatures (5 and 20°C, respectively). Changes in pH decrease, titratable acidity increase and redox potential increase as well as the viability of probiotics per 6 h intervals during an assumptive interrupted cold storage (24 h) were monitored. The survival of probiotics was strongly dependent on the storage temperature and remarkable viability loss occurred in room temperature compared to refrigerated storage. In addition, the survivability was dependent on probiotic strain. Among our experimental strains, *B. lactis *Bb-12 showed the less resistance to be stored at 20°C (24 h) and referring to the recommended minimum numbers of 10^7^ cfu mL^-^^1^, *L. rhamnosus *HN001 was the most suitable probiotic strain to be used in probiotic yogurts especially in countries having high possibility of cold chain interruption during storage.

## Introduction

Food industry companies have rather high expectations in food products that meet the consumers’ demand for a healthy life style. In this context, ‘functional foods’ play a specific role. These foods are not intended only to satisfy hunger and provide humans with necessary nutrients, but also to prevent nutrition-related diseases and increase physical and mental well-being of consumers (1-3). One of the most promising areas for the development of functional foods lies in the modification of gastrointestinal tract activity by the use of probiotics, prebiotics and synbiotics.

Probiotics are special types of live healthful bacteria or yeast which possess favorable impacts on animal and human host mainly via maintaining and/or improving microbial balance between harmful and beneficial microflora, especially in the intestine (4-6). They have an established role in reducing human illnesses, particularly gastrointestinal infections caused by deficient or compromised gut microflora. Other therapeutic functions are attributed to probiotics such as anti-cholesterol activity, alleviation of lactose intolerance symptoms, promotion of beneficial immune responses, antimicrobial impact, anti-high blood pressure effect and anticarcinogenic and anti-mutagenic activities (7-15). Recently, there have also been recent reports on the potential benefit of probiotics for human skin (16) and against colds and flu (17).

Probiotic microorganisms are common to be ingested through dairy products, mainly fermented milk products (5, 6). *Bifidobacterium *spp. and *Lactobacillus acidophilus *are by far the most important probiotics regularly added to the fermented milks (18). Among the dairy-fermented products, yogurt is the most popular one, and in Europe, the highest consumption of probiotic products is associated with probiotic yogurt (19).

Probiotics should be alive to an adequate number in order to exert their positive effects on the health of the host. This attribute is known as ‘viability’, namely the adequate number of live probiotic cells in a food product at the time of consumption (5). No general agreement has been reached on the recommended levels and the suggested levels ranged from 10^6^ cfu mL^-1^ (20) to over 10^7^ and 108 cfu mL^-1^ (5). However, it is generally recommended that the probiotic culture must be present in the product at minimum numbers of 10^7^ cfu mL^-1^ (21). These suggestions have been made to compensate for the possible decline in the concentration of the probiotic organisms during processing and storage of a probiotic product as well as passage through the upper and lower parts of the gastrointestinal tract. Numerous studies have demonstrated that probiotic strains grow poorly in milk, resulting in low final concentrations in yogurt and even the loss of the viability during prolonged and/or inappropriate storage conditions. Survival of these bacteria during shelf life and until consumption is therefore an important issue.

Various factors have been recognized to affect the viability of probiotic bacteria during storage of fermented dairy products such as pH, redox potential and acidity, buffering capacity, packaging, molecular oxygen and storage time and temperature (5, 22). Storage temperature has substantial impact on maintaining viability of probiotic bacteria and it is generally well-known that the probiotic yogurt must be kept under refrigerated storage (22- 24). However, mentioned product might be subjected to cold chain interruption for hours during industrial distribution, retailing and home storage. This leads to the question whether the number of probiotic bacteria declines to such an extent that there are not enough bacteria remaining in the product to be useful. Few studies have studied the effect of cold chain interruption during storage on the survivability of probiotics (25). In this study, the viability of probiotic bacteria in yogurts exposed to cold temperature interruption (stored at room temperature) was investigated.

## Experimental

*Starter cultures*

The DVS pouches of commercial lyophilized cultures including Y-type/yogurt bacteria (mixed culture of *Streptococcus thermophilus *and *Lactobacillus delbrueckii *ssp. *bulgaricus*) commercially known as YF-702 were supplied by Chr. Hansen (Horsholm, Denmark). Besides, a single probiotic culture containing *L. acidophilus *LA-5 (Chr. Hansen) or *Bifidobacterium lactis *Bb-12 (Chr. Hansen) or *L. rhamnosus *HN001 (Danisco, Copenhagen, Denmark) or *L. paracasei *Lpc-37 (Danisco) was used. These starter cultures are widely used by the dairy industry to produce fermented milk products. The cultures were maintained according to the manufacturer’s instructions, until used.

*Microbiological analysis*

Lactobacilli and bifidobacteria were enumerated selectively using MRS-bile agar (MRA agar: Merck, Darmstadt, Germany, and Bile: Sigma, Rede, USA) (Mortazavian *et al*. 2007a). The plates were incubated at 37°C for at least 72 h under both aerobiosis and anaerobiosis. Anaerobic condition was generated by using the Gas Pack system (Merck, Darmstadt, Germany).

Viability proportion index (VPI) of probiotic microorganism at the end of storage time were calculated as following (26, 27): VPI = Final cell population (cfu mL^-1^) / initial cell population (cfu mL^-1^).

*Sample preparation*

Yogurt milk with 12.0% dry matter was formulated using reconstituted skim milk powder. After heat treatment (90°C–15 min), cooling to inoculation temperature (40°C) and addition of yogurt starter culture, treatments were inoculated with different probiotic single strain cultures (10^6^ cfu mL^-1^). The treatments were incubated at 40°C until pH of 4.5 ± 0.02 was reached. The yogurts were stored for 24 h at two different temperatures including cold or room temperatures (5 or 20°C, respectively). Therefore, eight treatments were produced; namely yogurts containing *L. rhamnosus *HN001 stored at 5°C (RY-5) or 20°C (RY-20), *L. paracasei *Lpc-37 stored at 5°C (PY-5) or 20°C (PY-20), *L. acidophilus *La-5 stored at 5°C (AY-5) or 20°C (AY-20), and *B. Lactis *Bb-12 stored at 5°C (BY-5) or 20°C (BY-20). Changes in pH decrease, redox potential increase and titratable acidity increase as well as the viability of probiotics per 6-h intervals during 24 h of storage were monitored.

*Chemical analysis*

pH and redox potential values of the samples were measured at room temperature using a pH meter (MA235, Mettler, Toledo, Switzerland). The titratable acidity was determined after mixing 10 mL of sample with 10 mL of distilled water and titrating with 0.1 N NaOH using 0.5% phenolphthalein (26).

*Statistical analysis*

All results were an average of three replicate determinations and the significant differences among the means were analyzed using the two-way ANOVA test (based on the complete randomized design (full Factorial test design) from Minitab software (Version 13, 2002).

## Results and Discussion

*Changes in pH, titratable acidity and redox potential*

**Figure 1:**
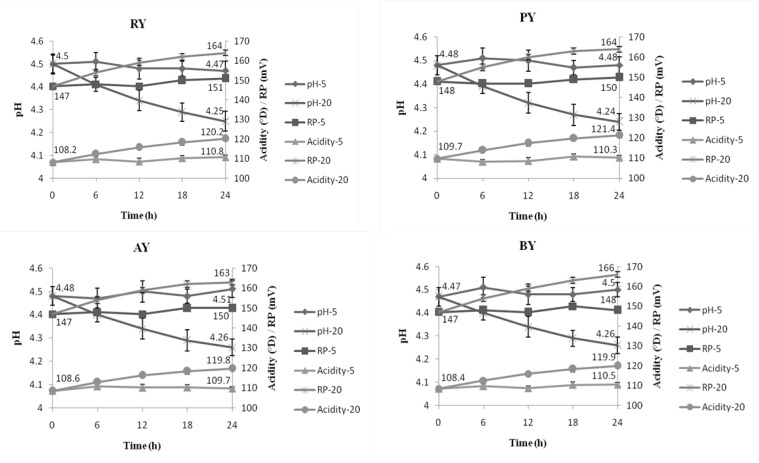
Changes in pH, titratable acidity and redox potential in treatments during storage (5 or 20°C). RY = *L. rhamnosus*, PY = *L. paracasei*, AY = *L. acidophilus*, BY = *B. Lactis*. The numbers ‘5’ and ‘20’ represent the storage temperature.

Figure 1 shows changes in pH, redox potential and acidity changes at every 6 h, among treatments stored at 20°C compared to the control (stored at 5°C). pH, titratable acidity and redox potential in treatments stored at 5°C were not significantly changed during 24 h of storage and were about 4.47, 110.8°D and 151 mV at the end of storage, respectively. Nevertheless, these values were approximately 4.25, 120.2°D and 164 mV in those stored at 20°C, respectively. As shown in Figure 1, pH decrease as well as titratable acidity and redox potential increase showed highest slopes at initial hours of storage than the later ones.

*Survival of probiotic microorganisms during storage*

Table 1 indicates viable counts of probiotic bacteria in different treatments during the storage time. Table 2 represents viability proportion index (VPI) in different treatments during this time. 

**Table 1:** Viable counts (log cfu mL^-1^) of probiotic bacteria in different treatments during storage time*.

**Treatments**	**Storage time (h)**				
	0	6	12	18	24
RY-5**	7.24^a^	7.24^a^	7.24^a^	7.24^a^	7.23^a^
RY-20	7.24^a^	7.21^ab^	7.17^b^	7.11^b^	7.02^c^
PY-5	7.20^a^	7.19^a^	7.20^a^	7.19^a^	7.19^a^
PY-20	7.20^a^	7.16^ab^	7.11^b^	7.02^c^	6.90^d^
AY-5	7.28^a^	7.29^a^	7.29^a^	7.28^a^	7.27^a^
AY-20	7.28^a^	7.22^a^	7.13^b^	7.03^c^	6.88^d^
BY-5	7.18^a^	7.18^a^	7.17^a^	7.17^a^	7.16^a^
BY-20	7.18^a^	7.11^b^	7.00^c^	6.84^d^	6.63^e^

* Means in the same row with different letters are significantly different (p < 0.05). ** Treatments stored at room temperature include: RY-20 = yogurts containing *L. rhamnosus*; PY-20 = yogurts containing *L. paracasei*; AY-20 = yogurts containing *L. acidophilus*; BY-20 = yogurts containing *B. Lactis*

**Table 2:** Viability proportion index (VPI) in different treatments during storage time (compared to the initial viable cell counts immediately after fermentation or the viable cell counts at the last hours of each 6-h storage interval).

**Treatments**	**VPI**_6_	**VPI**_12_		**VPI**_18_		**VPI**_24_	
	**h 0**	**h 0**	**h 6**	**h 0**	**h 12**	**h 0**	**h 18**
RY-5*	1.00	1.00	1.00	1.00	1.00	0.97	0.97
**RY-20**	0.93	0.85	0.90	0.74	0.87	0.60	0.81
**PY-5**	0.97	1.00	1.02	0.97	0.97	0.97	1.00
**PY-20**	0.91	0.81	0.88	0.65	0.81	0.50	0.76
**AY-5**	1.02	1.02	1.00	1.00	0.98	0.97	0.97
**AY-20**	0.86	0.70	0.81	0.56	0.79	0.39	0.70
**BY-5**	1.00	0.97	0.97	0.97	1.00	0.95	0.98
**BY-20**	0.84	0.66	0.78	0.45	0.69	0.27	0.60

* Treatments stored at room temperature: RY-20 = yogurts containing *L. rhamnosus*; PY-20 = yogurts containing *L. paracasei*; AY-20 = yogurts containing *L. acidophilus*; BY-20 = yogurts containing *B. Lactis*

According to Table 1, viable counts of probiotics in all treatments stored at 5°C had no significant changes during storage (p < 0.05). For example, the VPI (Table 2) for all strains at the end of storage ranged between 0.95-0.97. However, for treatments stored at 20°C, the viable counts of probiotics showed significant decrease during storage. This decline varied among probiotic species because of different sensitivity to environmental stresses such as low pH and high titratable acidity. Considering Table 2, the most survivability throughout the storage in treatments stored at 20°C belonged to *L. rhamnosus *HN001, *L. paracasei *Lpc-37, *L. acidophilus *LA-5 and *B. Lactis *Bb-12, respectively. *B. Lactis *maintained only 60% of its initial viable population at the end of storage, whilst this amount was 81% for *L. rhamnosus*. Scharl *et al*. (25) demonstrated that the number of living probiotic bacteria in yogurt decreased dramatically after exposure to room temperature. 

Considerable loss in viability of probiotics in room temperature could be attributed to increasing cell metabolism and death at higher temperatures (compared to refrigerated storage) as well as to the enhanced antagonistic impact of yogurt bacteria (especially *L. delbrueckii *ssp. *bulgaricus*) on probiotic bacteria. Yogurt bacteria can suppress probiotics during yogurt storage via ‘post-acidification’ process (28) which is noticeably intensified in temperatures of more than 5ºC. Within aforementioned process, not only increasing titratable acidity and decreasing pH but also formation of some metabolites such as hydrogen peroxide, short-chain fatty acids and bacteriocins are highly detrimental to probiotic cells (5). In all treatments exposed to room temperature, an increase in titratable acidity to 120.2°D during storage is the main evidence for post-acidification. Mortazavian *et al*. (29) reported that storage of ABY-type fermented milks at temperatures of more than 5ºC (8°C) led to a domination of *L. delbrueckii *ssp. *bulgaricus *and excessive post-acidification by this organism.

Considering Table 2, the rate of viability loss for each probiotic strain increased appreciably toward the end of storage time (hours 0, 6, 12 and 18 in VPI_6_^,^_12, 18 and 24_^,^ respectively; *e.g*., 0.93, 0.90, 0.87 and 0.81 for RY-20). The viable counts of probiotics were decreased by < 1 log cycle in all treatments (Table 1) and the count of > log 7 cfu mL^-1^ (minimum recommended level) in the treatments stored at room temperature was only observed for *L. rhamnosus *at the end of storage (24 h). *B. lactis *had the poorest viability and was able to maintain its survival higher than log 7 cfu mL^-1^ only for 12 h (room temperature). This time for *L. acidophilus *and *L. paracasei *was 18 h. Therefore, *L. rhamnosus *HN001 was the most suitable probiotic strain to use in probiotic yogurts especially in countries having high possibility of cold chain interruption during storage (after industrial dispatching) or in those that refrigeration facilities are absent.

## Conclusion

This study demonstrated that the survival of probiotic bacteria in commercially available yogurts is critically dependent on the conditions of how the products are stored. The survival of probiotics was strongly dependent on the storage temperature and remarkable viability loss occurred in room temperature. In addition, the decline in viability was dependent on the strain of probiotic. Among our experimental strains, *B. lactis *Bb-12 showed the less resistance to be stored at 20°C (24 h) and referring to the recommended minimum numbers of 10^7^ cfu mL^-1^, *L. rhamnosus *HN001 was the most suitable probiotic strain to be used in probiotic yogurts especially in countries having high possibility of cold chain interruption during storage (after industrial dispatching) or in those that refrigeration facilities are absent. However, the viability of *L. rhamnosus *HN001 for over 24 h storage of probiotic yogurt, utilization of other probiotic strains and effect of microencapsulation of probiotics on their viability during storage at room temperature should be investigated.
